# Toxic epidermal necrolysis co-existing with severe onset of ulcerative colitis—different condition or extraintestinal feature?—case report

**DOI:** 10.1007/s00384-015-2285-8

**Published:** 2015-06-24

**Authors:** Michał Aporowicz, Piotr Czopnik

**Affiliations:** Department and Clinic of General, Gastroenterological and Endocrine Surgery, Wroclaw Medical University, Marii Skłodowskiej–Curie 66, 50-369 Wrocław, Dolnośląskie Poland

Dear Editor:

We would like to report an interesting case of a man suffering from both ulcerative colitis (UC) and toxic epidermal necrolysis (TEN) at the same time. We are aware that such cases are already described in medical literature. However, previous articles perceive TEN as an adverse effect of derivatives of 5-aminosalicyclic acid (5-ASA), which are the foundation of conservative management of UC. In our patient, UC was diagnosed postmortem so he did not receive any drugs for UC at the time his cutaneous symptoms appeared. We believe it to be the first report of such co-existence without involvement of 5-ASA.

The described patient was a 49-year-old man with left kidney agenesia, suffering from type 2 diabetes and chronic constipation. Surgical history included appendectomy and laparotomy due to fecal obstruction of the sigmoid colon nine years before. His constant medications were as follows: acetylosalicylic acid 75 mg 1×, propranolol 40 mg 2×, glimepiride 4 mg 1×, metformin 1000 mg 3×, vinpocetine 10 mg 2×, piracetam 1200 mg 2×, and pridinol 5 mg 2×.

In late 2012, he developed slowly expanding bullae. Ambulatory management with glucocorticoid (i.v./i.m. dexametasone 4 mg) was administered but unsuccessful. Due to unknown etiology of his condition and ineffectiveness of empiric treatment, he was admitted in early 2013 to the Department of Dermatology of Wroclaw Medical University (WMU). Upon admission, multiple small and large bullae filled with serous fluid were present on all of the patient’s skin. Mucosae were not affected. Since day one of hospitalization, the patient had no bowel movement.

Series of laboratory, radiological, and pathomorphologic tests were performed. Laboratory tests revealed the following: (1) elevated inflammatory markers (WBC 28,400/μl, CRP 84.5 mg/l) and eosinophilia (11.2 %) in peripheral blood smear, (2) decreased level of total plasma protein (5.2 g/dl) and albumin (3.3 g/dl), (3) slightly decreased level of vitamin B12 and folic acid, but no anemia (RBC 5,670,000/μl, HGB 16.7 g/dl, HCT 46.6 %), (4) dyslipidemia (HDL cholesterol 23 mg/dl, triglycerides 240 mg/dl), and (5) bad metabolic control of diabetes (glycemia up to 459 mg/dl, HbA_1C_ 7.1 %, glycosuria 500 mg/dl). Tumor markers (CA15-3, CA19-9, CEA, PSA, AFP, and CA125) as well as virusological studies were negative: anti-HBe (−), anti-HBs level normal, anti-HCV (−), and anti-HBc (+). Chest X-ray showed no abnormalities.

Direct immunofluorescence (DIF) study of skin sample was performed. Linear concretions of C3c along the basement membrane zone (BMZ) and additionally sparse fine-grained concretions of IgM along BMZ were discovered. There were no IgG, IgA, or C1q concretions. The result of DIF study suggested the diagnosis of pemphigoid. Histopathological examination of skin sample was also performed. It revealed vast subepidermal bullae, which might have indicated pemphigoid.

After diagnosing pemphigoid, systemic treatment with sulfone (dapsone 50 mg, than 100 mg daily) and i.v. glucocorticoid (methylprednisolone 250 mg—2 doses at the fifth and ninth day) as well as local dressings (sulfathiazole with silver, hydrocortisone, oxytetracycline) were applied. Pain-management involved paracetamol and tramadol. Glycemia was controlled by metformin (1000 mg 3×) and glimepiride (4 mg); the latter was then switched to insulin (intermediate- and short-acting). Other medications taken by the patient before hospitalization were sustained.

At the eighth day of hospitalization, progression of cutaneous condition was observed. Extensive epidermal exfoliation, as in TEN, as well as new bullae involving about 60 % of the body area appeared. New lesions spread also to mucosae, which might correspond with clinical manifestation of TEN (Lyell syndrome). Dapsone and propranolol therapy, the most suspected of causing TEN, was discontinued. Second DIF result was identical. Immunosuppressant (cyclosporine 200–0–100 mg p.o., than 100–0–50 mg i.v.) and antibiotics (ceftriaxone 1000 mg 1× i.v.) were introduced, thus stopping the progression of lesions.

After nine days of hospitalization, the patient still had no bowel movement; in addition, he vomited several times. Consulting internist reinstated propranolol (20 mg 2×) and added metoclopramide (10 mg p.o. or i.v. 2–3×) and glycerol suppositories. Consulting anesthesiologist performed venous blood gases examination (pH 7.43, pCO_2_ 34.8 mmHg, pO_2_ 54.6 mmHg, sO_2_ 88.9 %). Consulting surgeon described the abdomen as non-tender, with no peritoneal signs, no masses, and present peristaltic sounds. Conservative treatment was recommended, consisting of strict diet, i.v. fluids, electrolytes supplementation, pantoprazole (40 mg 2×), metoclopramide (10 mg 3×), an enema, and abdomen X-ray in standing position.

Increase of abdomen circumference and symptoms of subacute bowel obstruction appeared. Prokinetic drugs, further enemas, and gastric tube were ordered. During the following days, patient’s condition did not improve. Abdomen CT showed distension of the colon and aroused a suspicion of fecalith in the sigmoid colon. Other organs showed no abnormalities.

Due to progression of symptoms of bowel obstruction, the patient was transferred to the Department of General Surgery of WMU at the 12th day of hospitalization and operated at the same day. Laparotomy revealed the distended small intestine with multiple adhesions, enormous distension of the entire colon (diameter about 15 cm) filled with feces as well as the thickened and inflamed colonic wall with no haustra. In spite of both features of toxic megacolon and irreversibility of colonic pathology, subtotal colectomy with terminal ileostomy was performed. After procedure, the patient developed acute circulatory and respiratory insufficiency and was transferred to the Department of Intensive Care of WMU. During the next several hours, symptoms of septic shock appeared.

In the Department of Intensive Care, multidisciplinary treatment was applied including the following: (1) supplementation of fluids and electrolytes, albumin infusions, (2) blood transfusions, (3) catecholamines, (4) management of acute renal failure (loop diuretics, discontinuation of cyclosporine, continuous venovenous hemodiafiltration (CVVHDF)), (5) antibiotics, (6) parenteral and enteral nutrition, (7) antithrombotic agents, (8) continuous insulin infusion, (9) dressings with potassium iodide and greasing agents. During several days, patient’s condition stabilized, requirement for vasopressors decreased, and hemodiafiltration was no longer necessary. After initial improvement, symptoms of septic shock reappeared and signs of circulatory failure and levels of inflammatory markers increased, despite antimicrobial treatment. At the 26th day of hospitalization, the patient developed multi-organ failure (MOF) and eventually died at the 27th day of hospitalization.

Histopathological examination of specimen revealed necrosis of the colon mucosa about 80 cm in length (total length 140 cm). Pathologist diagnosed active UC and chronic inflammation of the surrounding tissues and greater omentum as well as present nervous ganglia in the colon wall (that excluded potentially possible diagnosis of late Hirschprung disease). Immunohistochemical study was positive for S-100 marker.

UC may be accompanied by various pathologies of other organs, which are called extraintestinal complications. They occur in about 30 % of patients and manifest themselves in the joints, eyes, and skin. Cutaneous complication is divided into reactive, specific, nutrition-related, and others. Reactive complication includes erythema nosodum, pyoderma gangrenosum, aphthous ulcers of oral cavity, and necrotic vasculitis.

TEN is a severe pathology of the skin and mucosa. Erythema, bullae, and epidermal exfoliation are present. Severe TEN may be accompanied by inflammation of the mucosa, mostly in proximal part of the digestive tract. UC-like pathology was never described as a part of Lyell syndrome. Etiology of TEN is immunological, and in most cases, TEN itself is a reaction to infection or drug. Nonetheless, in some cases, the provoking factor remains unknown. The list of drugs that may induce TEN includes antibiotics (trimethoprim-sulfamethoxazole and other sulfonamides, aminopenicillins, cephalosporins, and quinolones), anti-convulsive drugs (carbamazepine, phenytoin, and phenobarbital), allopurinol, and II generation of NSAIDs (coxibs). Our patient did not receive any of the above prior to first skin lesions.

TEN, as well as its milder form, and Stevens–Johnson syndrome (SJS) have not been considered as being extraintestinal manifestations of UC before. Very few cases of co-existence of TEN and UC are described in the medical literature. The Medline database contains three such publications in English. In all three papers, treatment of UC with derivatives of 5-ASA was pointed out as a cause of TEN. None of them suggests a direct relation between these conditions nor treats TEN as an extraintestinal complication of UC. Appearance of TEN may be triggered by a lot of drugs, and the actual prevalence of TEN in patients treated with 5-ASA is very low.

The question remains whether cutaneous lesions were linked to digestive tract pathology in the case of our patient. We were not able to determine whether toxic megacolon developed as a complication of TEN or whether the patient had suffered UC before, which symptoms were masked by severe TEN. Dermatological symptoms may have also preceded development of severe onset of UC. Could TEN be viewed as an extraintestinal manifestation of UC or was this just a random coincidence and bullous lesions were a reaction to unknown antigen? Toxic megacolon might also have been a consequence of patient’s severe general condition or developed as the most severe form of mucosal lesions in TEN. Moreover, imposition of two severe conditions made both proper evaluation of digestive tract pathology and its treatment very difficult. The presented issue requires further observation. Perhaps collection of a greater number of individual cases in the future will allow us to describe the exact interference between UC and TEN.

Michał Aporowicz

Piotr Czopnik

